# Mi-RNA-888-5p Is Involved in S-Adenosylmethionine Antitumor Effects in Laryngeal Squamous Cancer Cells

**DOI:** 10.3390/cancers12123665

**Published:** 2020-12-07

**Authors:** Martina Pagano, Laura Mosca, Francesca Vitiello, Concetta Paola Ilisso, Alessandra Coppola, Luigi Borzacchiello, Luigi Mele, Francesca Pia Caruso, Michele Ceccarelli, Michele Caraglia, Giovanna Cacciapuoti, Marina Porcelli

**Affiliations:** 1 Department of Precision Medicine, University of Campania “Luigi Vanvitelli”, Via L. De Crecchio 7, 80138 Naples, Italy; martina.pagano@unicampania.it (M.P.); laura.mosca@unicampania.it (L.M.); francesca.vitiello@unicampania.it (F.V.); concettapaola.ilisso@unicampania.it (C.P.I.); alessandra.coppola@unicampania.it (A.C.); luigi.borzacchiello@unicampania.it (L.B.); giovanna.cacciapuoti@unicampania.it (G.C.); marina.porcelli@unicampania.it (M.P.); 2 Department of Experimental Medicine, University of Campania “Luigi Vanvitelli”, 80138 Naples, Italy; luigi.mele@unicampania.it; 3 Department of Electrical Engineering and Information Technology (DIETI), University of Naples “Federico II”, 80128 Naples, Italy; francescapia.caruso@gmail.com (F.P.C.); michele.ceccarelli@unina.it (M.C.); 4 Bioinformatics Lab, BIOGEM, 83031 Ariano Irpino, Italy; 5 Laboratory of Precision and Molecular Oncology, BIOGEM, 83031 Ariano Irpino, Italy

**Keywords:** S-Adenosylmethionine, laryngeal squamous cancer cells, apoptosis, autophagy, ER-stress, microRNA regulation, miR-888

## Abstract

**Simple Summary:**

Laryngeal Squamous Cell Carcinoma (LSCC) is a leading cause of cancer-related death with a strong interest in identifying and developing new treatments. MicroRNAs (miRNAs) have emerged as one of the most important determinants of neoplastic transformation and progression. miRNA modulation causes significant antitumor effects both in vitro and in vivo and miRNA regulation by natural compounds, represents a promising approach in the field of cancer research. S-Adenosylmethionine (AdoMet), a natural compound and a nutritional supplement, is well known for its antiproliferative and pro-apoptotic effects in many kinds of human tumors. Here, we report that AdoMet induces ER-stress and autophagy paralleled by miR-888-5p downregulation and *MYCBP* and *CDH1* increased expression in Laryngeal Squamous Cancer Cells (LSCC). This study contributes to understanding the mechanisms by which AdoMet exerts its effects in LSCC, suggesting the use of AdoMet as an attractive miRNA-mediated chemopreventive and therapeutic strategy against cancer.

**Abstract:**

(1) Purpose: The methyl donor S-Adenosylmethionine (AdoMet) has been widely explored as a therapeutic compound, and its application-alone or in combination with other molecules-is emerging as a potential effective strategy for the treatment and chemoprevention of tumours. In this study, we investigated the antitumor activity of AdoMet in Laryngeal Squamous Cell Carcinoma (LSCC), exploring the underlying mechanisms. (2) Results: We demonstrated that AdoMet induced ROS generation and triggered autophagy with a consistent increase in LC3B-II autophagy-marker in JHU-SCC-011 and HNO210 LSCC cells. AdoMet induced ER-stress and activated UPR signaling through the upregulation of the spliced form of XBP1 and CHOP. To gain new insights into the molecular mechanisms underlying the antitumor activity of AdoMet, we evaluated the regulation of miRNA expression profile and we found a downregulation of miR-888-5p. We transfected LSCC cells with miR-888-5p inhibitor and exposed the cells to AdoMet for 48 and 72 h. The combination of AdoMet with miR-888-5p inhibitor synergistically induced both apoptosis and inhibited cell migration paralleled by the up-regulation of *MYCBP* and *CDH1* genes and of their targets. (3) Conclusion: Overall, these data highlighted that epigenetic reprogramming of miRNAs by AdoMet play an important role in inhibiting apoptosis and migration in LSCC cell lines.

## 1. Introduction

Laryngeal squamous cell carcinoma (LSCC) represents approximately one-third of all head and neck squamous cell carcinoma (HNSCC) and, due to its highly aggressive features, is considered a major cause of cancer-related deaths, with more than 150,000 new cases diagnosed annually and presently is the only tumor with an increase in mortality during the time [1,2]. Hereditary factors, alcohol, and tobacco consumption are well-known risk factors for the development of LSCC, and more recently, viral infections, such as human papilloma virus, have been implicated in the pathogenesis of an HNSCC subgroup [3,4]. Over the past three decades, LSCC treatment has changed drastically, moving from the classic surgical approach to the combined multidisciplinary treatments associated with concomitant or sequential chemotherapy and radiotherapy [1,2]. Despite the progress made in treatment regimes, the 5-year mortality rate has not changed, remaining around 50%. Both high toxicity, induced by the drugs used for the treatment of LSCC, and the occurrence of drug resistance mechanisms, are likely responsible for this detrimental effect. In this light, there is a strong interest in identifying and developing new LSCC treatments, taking advantage on the combination of natural compounds and cytotoxic agents, in order to improve therapeutic efficacy by reducing side effects and overcoming drug resistance mechanisms.

Among natural compounds, the role and therapeutic potential of S-adenosyl-L-methionine (AdoMet, also known as SAM or SAMe) to treat several human diseases, such as depression, liver illnesses, osteoarthritis, and others is already well known in the literature [5,6,7]. It is interesting to note that AdoMet is available as a dietary supplement and pharmaceutical preparation of this compound can be performed in intravenous, intramuscular, and oral forms. Reviews of clinical studies indicate that, at pharmacological doses, AdoMet has a low incidence of side effects with an excellent record of tolerability.

In last decade, growing evidence, accumulated in literature, describe the effects of AdoMet in pleiotropic biological processes, including the regulation of cell growth, apoptosis, autophagy and inhibition of invasion and metastatic spread in different kind of human cancers [5,8,9,10,11,12,13,14,15,16,17]. It is widely reported that AdoMet exerts its antitumor action through different biological pathways depending on cancer cell-type [5,8,10,11,12,13,14,17].

It was reported, indeed, that in pancreatic cancer, AdoMet enhanced the anti-metastatic effect of gemcitabine through the inhibition of the JAK2/STAT3 signaling pathway [17]. In human cervical cancer HeLa cells, the combination treatment with AdoMet and selenium compounds enhanced the anti-metastatic effect by affecting ERK and AKT signaling pathways [12]. We have reported the synergistic effects of AdoMet with doxorubicin in regulating hormone-dependent breast cancer cell line CG5 proliferation by inducing the activation of the Fas/FasL signaling pathway [10]. Moreover, we have also evaluated the combination effect of AdoMet and chloroquine (CLC), an autophagic inhibitor, in the MCF-7 breast cancer cell line, showing that autophagy block was synergistic in inducing both growth inhibition and apoptosis [18]. More recently, we have demonstrated that AdoMet is able to sensitize HNSCC cells to the pro-apoptotic effect induced by cisplatin [15]. We have also demonstrated that the combination of AdoMet and cisplatin synergistically inhibited HNSCC cell migration [16]. Our previous studies on HNSCC allowed us to consider AdoMet as a potential candidate for drug development against HNSCC, based on its ability to modulate cancer cell growth and survival through the simultaneous regulation of multiple signaling pathways [15,16].

Recent studies have reported that the action of AdoMet can also be mediated by the regulation of expression of microRNAs (miRNAs). miRNAs are a class of small non-coding endogenous RNAs (about 20-22 nucleotides) that play an important role in the regulation of the expression levels of various mRNAs [19]. miRNAs have a significant role in carcinogenesis of head and neck cancers, acting either as oncogenes to promote cancer development and invasion or tumor suppressors by participating in the modulation of head and neck cell proliferation, differentiation, migration and invasion [20].

Therefore, the modulation of miRNA expression is emerging as a potential therapeutic target in many human cancers. In a recent work, it was demonstrated that, in hepatocellular carcinoma, the reduction in the expression of methionine adenosyltransferase genes, coding the enzyme that synthesizes AdoMet can be attributed to the regulation of miRNAs, resulting in decreased AdoMet levels and the deregulation of signal transduction pathways correlated to methionine metabolism and methionine adenosyltransferase activity [21]. In MCF-7 breast cancer cells, we have demonstrated that AdoMet induces apoptosis and autophagy by the regulation of the expression of miR-34a, miR-34c and miR-486-5p [22].

Although in recent years growing evidence has accumulated in the literature on the anti-proliferative and pro-apoptotic effects of AdoMet in different cancer cells, few data are actually available on the molecular mechanisms underlying the anticancer effect of AdoMet in HNSCC [15,16] and the modulation of miRNA expression by AdoMet in LSCC has never been studied before.

Here, we explored the pro-apoptotic mechanism of AdoMet in LSCC lines JHU-SCC-011 and HNO210. Moreover, to get new insight into the molecular mechanisms underlying the antitumor activity of AdoMet, we studied the AdoMet-induced modulation of miRNA expression profile. In particular we identified and investigated the AdoMet-induced miR-888 modulation in these two LSCC cell lines and its underlying mechanism of action.

## 2. Results

### 2.1. AdoMet Triggered Autophagy in LSCC

**Figure 1:**
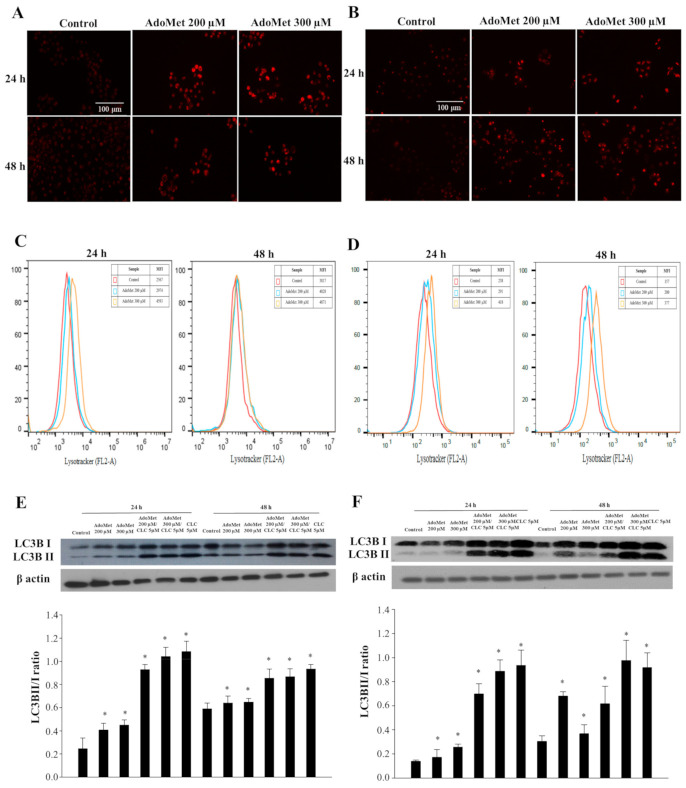
Effects of AdoMet on autophagic process in LSCC. Representative images of LTR staining of (**A**) JHU-SCC-011 and (**B**) HNO210 cells treated, or not (Control), with 200 and 300 μM AdoMet for 24 and 48 h analyzed by fluorescence microscopy. (**C**) JHU-SCC-011 and (**D**) HNO210 cells were treated, or not (Control), with the same concentrations of AdoMet at indicated times and flow cytometry analysis was performed. At least 2 × 10^4^ events were acquired in log mode. For the quantitative evaluation of LTR, FlowJo software was used to calculate median fluorescence intensities (MFI) by the formula (MFI-treated/MFI-control). Analysis was carried out by triplicate determination on at least three separate experiments. Western blot assay of (**E**) JHU-SCC-011 and (**F**) HNO210 cells for the expression of autophagy-related markers LC3B. Cells were cultured in medium, supplemented, or not (Control), with 200 or 300 μM AdoMet for 24 and 48 h, alone or in combination with 5 μM CLC. Graphs show the densitometric intensity of LC3B-II/I bands ratio. The protein intensities were expressed as arbitrary units (* *p* < 0.05 versus control cells). For the equal loading of protein in the lanes, β-actin was used. The experiments were repeated at least three times and always gave similar results. Uncropped images of Western blots are reported in Figure S1.

We previously found that 300 μM AdoMet induced apoptosis after 72 h by activating the caspase-dependent mechanism paralleled by increased Bax/Bcl-2 ratio [15]. To further investigate the mechanisms of anti-cancer activity of AdoMet, we evaluated the activation of the autophagy process in LSCC. We firstly analyzed autophagosome formation after staining with the vital dye LysoTracker-Red (LTR), a fluorescent probe for the labeling and tracking of acidic organelles in living cells, including autophagosome and autolysosome structures [23,24]. The treatment of JHU-SCC-011 (Figure 1A) and HNO210 (Figure 1B) cells with 200 and 300 μM AdoMet caused an increased formation of red dotted acidic vacuoles in comparison to untreated cells after 24 and 48 h. The quantitative analysis by flow cytometry of AdoMet-treated cells demonstrated a significant increase in the autophagic flux either in JHU-SCC-011 (Figure 1C) or HNO210 (Figure 1D) cells if compared to control, indicating the occurrence of a dose- and time-dependent autophagy. To confirm the induction of the autophagic process by AdoMet, we detected by Western blot the levels of LC3B, one of the main autophagy markers. AdoMet induced a concentration-dependent accumulation of LC3B-II protein (Figure 1E,F). Increased LC3-II levels correlated to the extent of autophagosome formation and was associated with either enhanced autophagosome synthesis or reduced autophagosome turnover. Therefore, to better interpret changes in levels of processed LC3-II, the cells were treated with AdoMet alone or in combination with CLC, the main lysosomal protease inhibitor. After 24 and 48 h of treatment with AdoMet, LC3B-II/I protein ratio was significantly increased, indicating that the further accumulation of LC3B-II in presence of AdoMet and CLC was due to the enhancement of autophagic flux rather than the inhibition of autophagosome degradation (Figure 1E,F). Overall, these results clearly demonstrated that AdoMet induced autophagy in JHU-SCC-011 and HNO210 LSCC cells.

### 2.2. AdoMet Promoted ER-Stress and Related UPR in LSCC

**Figure 2:**
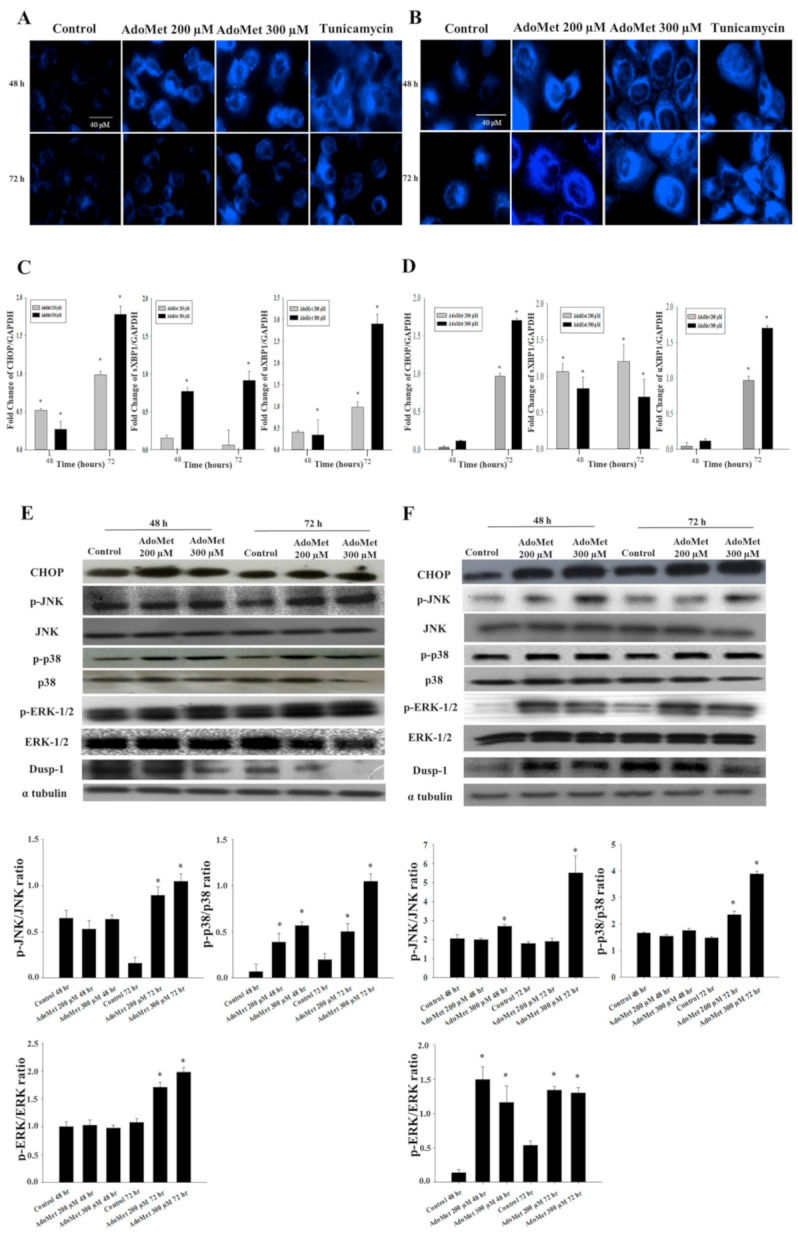
Effect of AdoMet on ER stress and related UPR in LSCC. (**A**) JHU-SCC-011 and (**B**) HNO210 cells were treated, or not (Control), with 200 and 300 μM AdoMet for 48 and 72 h and then were incubated with ER-tracker and analyzed by fluorescence microscopy. Tunicamycin was used as positive control. Total-RNA of (**C**) JHU-SCC-011 and (**D**) HNO210 cells was extracted and cDNA was synthesized by qRT-PCR, to investigate the transcription of several key proteins involved in ER-stress. The graphs show the fold change in CHOP, sXBP1, and uXBP1 normalized to GAPDH mRNA and compared to untreated cells. Data represent the average of three independent experiments. Bars, SD. * *p* < 0.05 versus control untreated cells. The expression levels of CHOP and of phosphorylated and total isoforms of JNK, p38, ERK 1/2, and Dusp-1 were detected by Western blot analysis using the total cell lysates of (**E**) JHU-SCC-011 and (**F**) HNO210 cells. The housekeeping protein, α-tubulin, was used as loading control. Graphs show the densitometric intensity of p-JNK/JNK, p-p38/p38, and p-ERK/ERK ratio. The intensities of signals are expressed as arbitrary units. * *p* < 0.05 versus control untreated cells. The images are representative of three immunoblotting analyses obtained from at least three independent experiments. Uncropped images of Western blots are reported in Figure S2.

ER stress has been identified as a key player in drug-induced apoptosis regulation [25]. Therefore, we evaluated AdoMet’s effect on ER-stress and the related unfolded protein response (UPR). JHU-SCC-011 and HNO210 cells were treated with 200 and 300 μM AdoMet for 48 and 72 h. The shape of treated cells was then evaluated by fluorescence staining using ER-Tracker, a vital dye that accumulates into ER, utilizing tunicamycin, a known inducer of ER stress, as a positive control. AdoMet increased the fluorescence of cells compared to untreated cells (Figure 2A,B), paralleled by morphological changes in the ER, providing a first indication that AdoMet was able to trigger ER-stress in LSCC cell lines.

To support the effectiveness of AdoMet as an ER-stress inducer, we analyzed the activation of relevant ER-stress markers, and their downstream signaling molecules. The quantitative analysis obtained by qRT-PCR experiments reported in Figure 2C,D showed that, in both JHU-SCC-011 and HNO210 cells AdoMet increased in a dose- and time-dependent manner the mRNA levels of CCAAT-enhancer-binding protein homologous protein (CHOP), a crucial factor that mediates ER-stress induced apoptosis [26]. The AdoMet-induced upregulation of CHOP observed at translational level was then confirmed by Western blot analysis, which evidenced an increase in the protein in both LSCC cell lines (Figure 2E,F). Then, we examined uXBP1, a transcription factor that, during ER stress, is activated by splicing into a more efficiently translated form. As found by qRT-PCR, AdoMet induced a significant increase in mRNA levels of uXBP1 and its spliced form sXBP1, which appeared more evident after 72 h of treatment.

The identification of numerous points of cross-talk between the UPR and mitogen activated protein kinase (MAPK) signaling pathways has contributed to a better understanding of their role in the response to ER stress [15]. Some MAPK pathways, such as p38 and JNK, are activated in response to ER stress and are part of the UPR process [27,28]. Therefore, we investigated whether the ER-stress triggering in LSCC upon AdoMet treatment occurred together with the induction of MAPK signaling. AdoMet upregulated the phosphorylated forms of ERK, JNK, and p38-MAPK in a time-dependent manner (Figure 2E,F). DUSP-1 is a dual-specificity phosphatase that selectively dephosphorylates MAPKs and primarily JNK [29,30]. When DUSP-1 increases, JNK activation is counteracted, which consequently protects tumor cells from JNK-induced apoptosis. Based on these considerations, we examined DUSP-1 protein level in both LSCC cell lines in response to AdoMet. AdoMet remarkably decreased the level of DUSP-1 in a concentration-dependent manner, as evaluated with Western blotting (Figure 2).

These data suggest that the induction of ER stress and the related UPR represent crucial events in AdoMet-induced apoptotic process in LSCC and that MAPKs contribute to the regulation of this cell death mechanism.

### 2.3. AdoMet Induced ROS Production in LSCC

Overproduction of reactive oxygen species (ROS) caused damage to the cells and was involved in the regulation of a variety of cellular processes, including autophagy, apoptosis, mitochondrial dysfunction, ER-stress and DNA damage [31]. Recently, it was reported that ROS generation induced by various antitumor agents is partly linked to ER-stress activation [31], indicating that there is a mutual relationship between ER stress and ROS accumulation.

**Figure 3:**
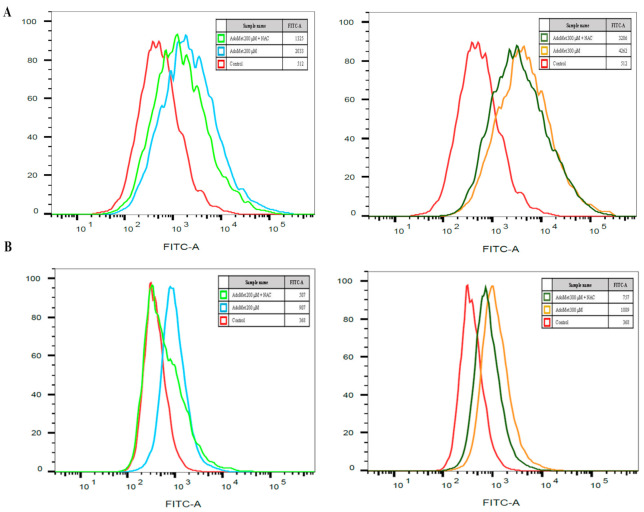
Effect of AdoMet on ROS accumulation in LSCC. (**A**) JHU-SCC-011 and (**B**) HNO210 cells were treated, or not (Control), with 200 and 300 μM AdoMet for 72 h with or without pretreatment with 5 mM NAC and then subjected to flow cytometry to measure ROS levels. FACS analysis was performed using 2′,7′-dichlorofluorescein diacetate (DCF-DA) as a substrate. For the quantitative evaluation of ROS, FlowJo software was used to calculate median fluorescence intensities (MFI) by the formula (MFI-treated/MFI-control). Analysis was carried out by triplicate determination on at least three separate experiments.

We have evaluated the levels of ROS in LSCC cell lines after the treatment with AdoMet. JHU-SCC-011 and HNO210 cells were treated with 200 and 300 μM AdoMet for 72 h and the intracellular ROS levels were quantitatively evaluated by flow cytometry using DCF-DA-based assay, a cell permeable, non-fluorescent precursor of DCF widely employed as an intracellular probe for ROS production. We found that about a 4- and 8-fold increase in ROS levels was found in JHU-SCC-011 after 72 h treatment with either 200 or 300 μM AdoMet, respectively, and about a 2.5- and 3-fold in HNO210 cells, respectively (Figure 3). This effect was antagonized by pretreatment with the ROS scavenger NAC, confirming the causative link between AdoMet and oxidative stress in LSCC cells. Interestingly, AdoMet-induced apoptosis was not reversed in NAC-pretreated cells, supporting the hypothesis that the activation of ER-stress could serve as a mechanism to trigger the apoptotic response of LSCC cells to AdoMet and that ROS generation could take place as a side effect induced by the mechanism of cell death.

### 2.4. AdoMet Changed miRNA Expression Profile in LSCC

MiRNA dysregulation plays an important role in tumorigenesis. To obtain new information on the molecular mechanisms underlying AdoMet anti-cancer activity in LSCC, we carried out miRNA expression profile of JHU-SCC-011 cells after treatment with 300 μM AdoMet for 72 h using a 384-well TaqMan Array CARD. Sixteen miRNAs were differentially expressed in AdoMet-treated cells if compared to control samples.

Among them, we focused our attention on miRNAs known to be associated with the regulation of the main pathways of cell death and proliferation, such as miR-187, miR-491-3p, miR-618 and miR-888-5p. Among these miRNAs, only miR-888-5p, whose expression levels were remarkably decreased over the control in TaqMan Array CARD (−2.6-fold), was validated by qRT-PCR analysis in JHU-SCC-011 and HNO210 cells. As shown in Table 1, miR-888-5p expression was 7.6-fold and 1.9-fold downregulated by AdoMet in JHU-SCC-011 and HNO210 cells, respectively, suggesting that AdoMet was able to reprogram non-coding miRNA expression in LSCC.

### 2.5. AdoMet and miR-888-5p Inhibitor Enhanced the Pro-Apoptotic Effect of AdoMet in LSCC

**Figure 4:**
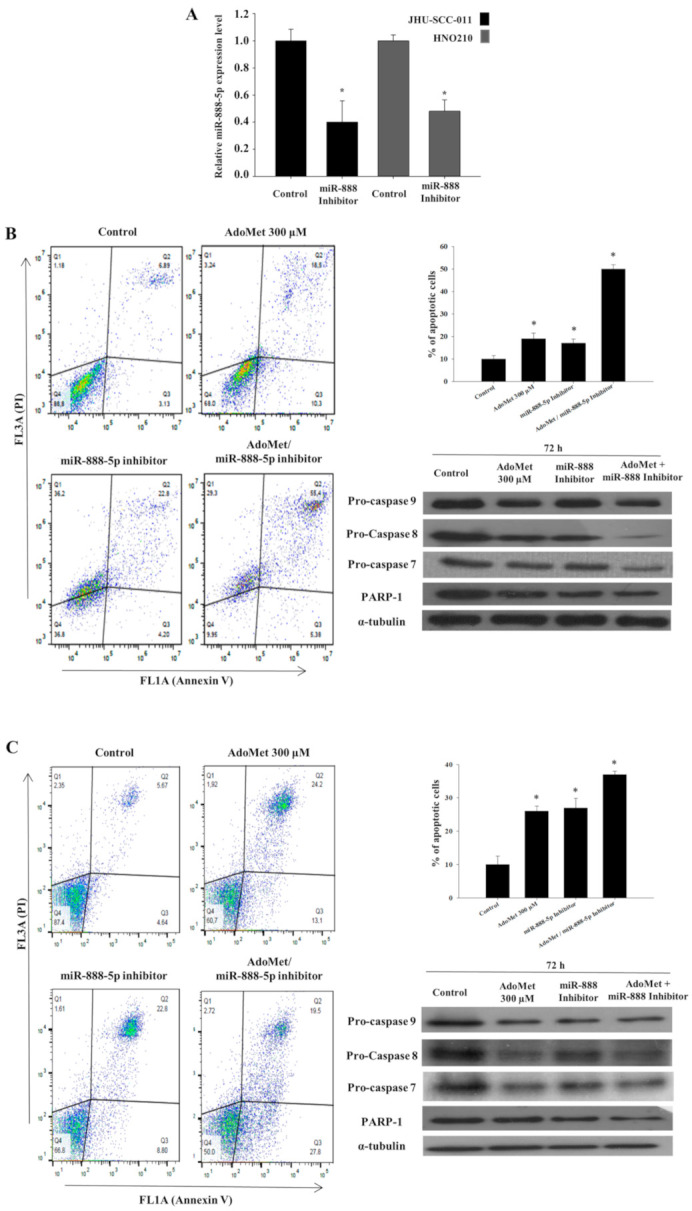
Effect of AdoMet and miR-888-5p inhibitor combination on apoptotic process in LSCC. (**A**) Effects of 50 nM miR-888-5p inhibitor transfection on the expression of miR-888-5p expression evaluated on JHU-SCC-011 and HNO210 cell lines. (**B**) JHU-SCC-011 and (**C**) HNO210 cells were transfected with 50 nM miR-888-5p inhibitor, supplemented or not (Control) with 300 μM AdoMet for 72 h. Apoptosis was evaluated by FACS analysis. Representative dot plots of both Annexin V-FITC and PI-stained cells. The different quadrants report the percentage of cells: viable cells, lower left (Q4); early apoptotic cells, bottom right (Q3); late apoptotic cells, top right (Q2); non-viable necrotic cells, upper left (Q1). For each sample, 2 × 10^4^ events were acquired. Analysis was carried out by triplicate determination of at least 3 separate experiments. In the histograms, the percentages of cells in the Q2 and Q3 quadrants evaluated by the FACS analysis were reported. Data represent the average of three independent experiments. The expression levels of pro-caspase 9, pro-caspase 8, pro-caspase 7, PARP were detected by Western blot analysis using the total cell lysates of (**B**) JHU-SCC-011 and (**C**) HNO210 cells. For the equal loading of protein in the lanes, α-tubulin was used. The images are representative of three immunoblotting analyses obtained from at least three independent experiments. Uncropped images of Western blots are reported in Figure S4.

To investigate the biological role of miR-888-5p in AdoMet-treated cells, we performed transfection experiments with the miR-888-5p inhibitor and evaluated the modulation of apoptosis. Firstly, we demonstrated that the miR-888-5p inhibitor transfection effectively downregulated miR-888-5p transcriptional levels by qRT-PCR (Figure 4A).

The transfection with 50 nM miR-888-5p inhibitor induced apoptosis in about 17% and 19% of JHU-SCC-011 and HNO210 cells, respectively (Figure 4B,C), suggesting that miRNA-888-5p acts as oncogenic miRNA in LSCC. In agreement with our previous results [15], about 19% of JHU-SCC-011 and 26% of HNO210 cell population, respectively, underwent apoptosis (Figure 4A,B, respectively) after treatment for 72 h with 300 μM AdoMet. Interestingly, we found that the combination of AdoMet and the miR-888-5p inhibitor increased apoptotic cell death up to about 40% and 37% in JHU-SCC-011 and HNO210 cells, respectively (Figure 4B,C). Conversely, no cell death was detected after treatment with scramble negative control (Figure S3), providing evidence that the effects evaluated on apoptosis in our experimental model are dependent on miR-888-5p inhibition. The pro-apoptotic effect of AdoMet and the miR-888-5p inhibitor was mediated by caspase-dependent mechanisms, as demonstrated by Western blot analysis that evidenced the decrease in pro-caspases 9, 8, and 7 and the cleavage of PARP-1 (Figure 4). Notably, similarly to the results obtained with FACS analysis, the activation of caspases and the degradation of PARP-1 were potentiated by the combination of AdoMet and miR-888-5p. All the data above provided evidence that the downregulation of the oncogenic miR-888-5p could represent one of the targets of AdoMet involved in apoptosis occurrence in LSCC.

### 2.6. AdoMet and miR-888-5p Inhibitor Affect EMT in LSCC

**Figure 5:**
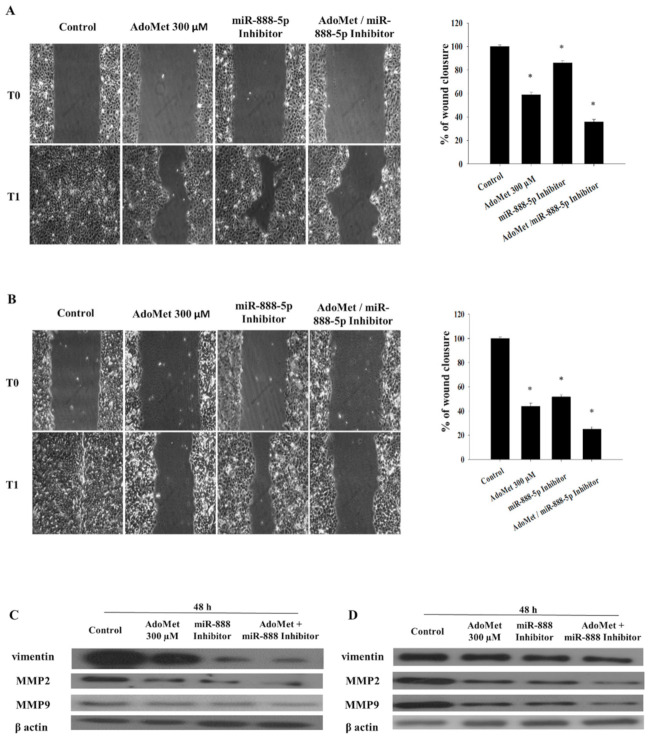
Effect of AdoMet and miR-888-5p inhibitor combination on cell migration in LSCC. Confluent monolayers of (**A**) JHU-SCC-011 and (**B**) HNO210 cells treated or not (Control) with 300 µM AdoMet and miR-888-5p-inhbitor alone or in combination for 48 h, were scratched with a micropipette tip and snapshot pictures were captured by microscope to check for wound closure. Pictures of the wounds corresponding to time zero (T0) and 24 h (T1) from the scrape in both cell lines are reported. Histograms show the quantification of wound area calculated as a percentage of the control using Image J software (U.S. National Institutes of Health, Bethesda, MD, USA). Data represent the average of three independent experiments. The means and SD are shown. * *p* < 0.05 versus control. The expression levels of vimentin, MMP2 and MMP9 were detected by Western blot analysis using the total cell lysates of (**C**) JHU-SCC-011 and (**D**) HNO210 cells. For the equal loading of protein in the lanes, β-actin was used. The images are representative of three immunoblotting analyses obtained from at least three independent experiments. Uncropped images of Western blots are reported in Figure S6.

To additionally investigate both the mechanism of anticancer activity of AdoMet in LSCC and the possible involvement of AdoMet-induced miR-888-5p downregulation, we investigated the effect of AdoMet and the miRNA-888-5p inhibitor, alone and in combination, on the migratory power of JHU-SCC-011 and HNO210 cells by wound healing assay, monitored for 24 h. We found that cell exposure to 300 µM AdoMet for 48 h caused about 59% and 44% wound closure in JHU-SCC-011 and HNO210 cells, respectively, compared to untreated cells (Figure 5A,B). A comparable inhibition of the wound healing was observed in both LSCC cell lines transfected with miR-888-5p inhibitor. Interestingly, combined treatments significantly increased the inhibitory effect of the single compound in both cell lines leading to wound closure values of 36% and 25% in the JHU-SCC-011 and HNO210 cells respectively, compared to the control (Figure 5). To verify the miR-888-5p inhibitor impact on migratory power of LSCC, we demonstrated that no significant changes were detected on cell migration after transfection with negative scramble control (Figure S5). To confirm the results of wound healing assay and to investigate whether the suppression of LSCC cell migration upon treatments with AdoMet and miRNA-888-5p inhibitor was associated with epithelial-mesenchymal transition (EMT), we analyzed by Western blot the expression patterns of the main migration markers characterizing the EMT process and migration-related protein markers, including vimentin, MMP2 and MMP9 (Figure 5C,D).

The analysis showed a marked decrease in vimentin, MMP2 and MMP9 levels, in the JHU-SCC-011 and HNO210 cells exposed to AdoMet and miR-888-5p inhibitor, alone or in combination for 48 h, as compared to the control.

These findings suggest that the combination of AdoMet and the miR-888-5p inhibitor was more effective in reducing cell migration than single agents, suggesting that the antitumor properties of AdoMet in LSCC can be explained, at least in part, by the modulation of EMT-suppressive miR-888-5p.

### 2.7. AdoMet Regulates the Expression of MYCBP and CDH1 by the Downregulation of miR-888-5p

**Figure 6:**
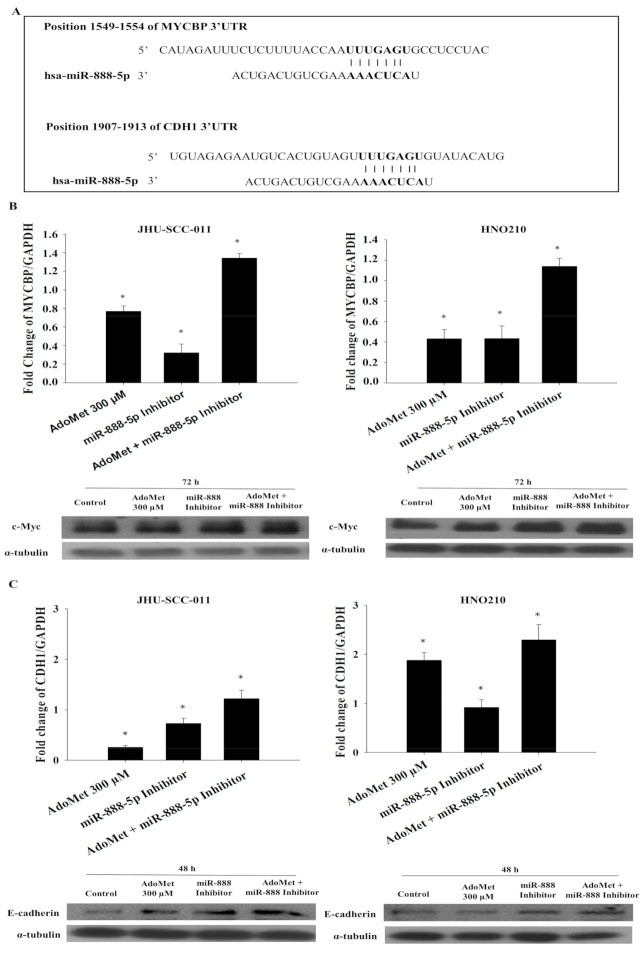
Effect of AdoMet and miR-888-5p inhibitor on *MYCBP* and *CDH1* expression. (**A**) Alignment of miR-888-5p with MYCBP or CDH1 3′UTR obtained from miRNA-mRNA integration analysis using the TargetScan microRNA target prediction software. (**B**,**C**) Cells were transfected with miR-888-5p inhibitor in the presence or not (Control) of 300 μM AdoMet for 48 and 72 h. Total-RNA of JHU-SCC-011 and HNO210 cells was extracted and cDNA was synthesized by qRT-PCR, to analyze the transcriptional level of predicted target. The graphs show the fold change (**B**) of MYCBP or (**C**) CDH1 in the different experimental conditions normalized to GAPDH mRNA and compared to untreated cells. JHU-SCC-011 and HNO210 cells were transfected with miR-888-5p inhibitor, in the presence, or not (Control), of 300 μM AdoMet for 48 h the detection of E-cadherin and for 72 h of c-MYC. Then, cell lysates were subjected to SDS-PAGE, incubated with antibodies against the indicated proteins and analyzed by Western blotting. The housekeeping protein, α-tubulin, was used as a loading control. Data represent the average of three independent experiments. Bars, SD. * *p* < 0.05 versus control untreated cells. Uncropped images of Western blots are reported in Figure S8.

To identify potential mRNA targets of miR-888-5p, we used two distinct computational algorithms, miRNA target prediction bioinformatic TargetScan [32] and miRBase software [33], finding that both programs indicated a set of common target genes. We then focused our attention on proteins that could be involved in AdoMet-mediated pathways including FAS receptor, the mitochondrial transporter SLC25C, metallopeptidase inhibitor, metallopeptidase inhibitor 4, E-cadherin (CDH1) and c-Myc binding protein (MYCBP) (Figure 6A). Although the expression of all these targets was increased by AdoMet and miR-888-5p treatment; only *MYCBP* and *CDH1* expression was enhanced by the combination of AdoMet and miR-888-5p inhibitor, indicating a functional relationship between the two compounds.

The qRT-PCR analysis highlighted that, in both LSCC cell lines, the combination of 300 µM AdoMet and miR-888-5p inhibitor enhanced the expression of MYCBP and CDH1 mRNA after 72 h and 48 h, respectively; much more than AdoMet or the miR-888-5p inhibitor alone (Figure 6B,C). Conversely, the qRT-PCR analysis of MYCBP and CDH1 mRNA, after miR-888-5p mimic transfection, alone or in combination with AdoMet after 72 h and 48 h, respectively, showed a decrease in miR-888-5p target levels (Figure S7).

Confirming the results of qRT-PCR, Western blot analysis showed that AdoMet and the miR-888-5p inhibitor individually increased the expression of c-Myc and E-cadherin in both LSCC cell lines and that their combined treatment was more effective than the single treatment (Figure 6B,C). Unlike CDH1, whose connection with miR-888-5p has been deeply evidenced in literature [34], no data are reported about *MYCBP* gene. Thus, to confirm the relevance of miR-888-5p as post-transcriptional regulator of *MYCBP* gene, we analyzed miRNA levels and gene expression profiles in a TCGA cohort of head and neck squamous carcinoma (HNSC) tumor patients. The Spearman correlation (R^2^ = −0.13, *p*-value = 0.004) showed an inverse trend of miR888 and MYCBP expression in this patient cohort.

The obtained results suggest that *MYCBP* and *CDH1* are direct targets of miR-888-5p and their upregulation by AdoMet may contribute to the antiproliferative activity of the sulfonium compound.

## 3. Discussion

LSCC, with highly invasive and metastatic malignant behavior, represent the second most prevalent malignancy of the upper aerodigestive tract. Despite recent improvement in oncological and surgical treatment, the prognosis remains extremely poor [35] with the overall 5-year survival rate at about 60%. Surgery, radiation, chemotherapy, targeted treatments and immunotherapy, are commonly used to treat laryngeal cancer. However, these treatment types may cause different side effects, and chemotherapy-based regimens appear to have reached a therapeutic plateau [2]. Therefore, a better understanding of molecular mechanisms underlying LSCC is particularly necessary to improve the prognosis of LSCC patients.

New advances in anticancer drug discovery using natural compounds have been made in the last few years in order to improve therapeutic treatment options. AdoMet, an important and naturally occurring sulfonium compound, in addition to the well-known biological functions, exerts antiproliferative action in many types of human cancer cells [5,8,10,11,12,13,14,15,17,18]. The sulfonium compound is widely used in the treatment of several diseases and is introduced as a food supplement, as the absence of the common contraindications induced by the most widely used chemotherapy drugs is well demonstrated. Recently, growing scientific interest is focused on identifying the biological mechanisms and the signal transduction pathways related to the chemopreventive activities of AdoMet.

Recent studies reported AdoMet as a modulator of miRNA expression, resulting in the impairment of signal transduction pathways correlated to apoptosis, cell cycle and autophagy [19]. The regulation of miRNAs by natural compounds represents a promising approach in the field of cancer research. A single miRNA can influence the expression of several targets for degradation or translation repression, consequently their dysregulation can lead to human diseases, including cancer. Differentially expressed miRNAs were reported in diverse cancer types, such as breast [36], lung [37], prostate [38], and head and neck cancer [39]. The interest in the study of therapeutic strategies that act on miRNAs is linked to the fact that, by intervening therapeutically on a single molecule, it is possible to obtain multiple effects on the gene expression. A preliminary and very important phase in the development of new treatments is the study of the mechanism triggered by the antitumor drugs in cancer cells.

In the present study, we investigated AdoMet anticancer activities in LSCC, providing experimental evidences on the underlying mechanisms as well as at the regulation of miRNA expression.

AdoMet induced ER-stress and activated the UPR. Although the activation of the ER-stress response leads to adaptations that may aid cell survival, it is well known that, under severe and prolonged ER-stress conditions, where the cells fail to restore ER homeostasis, the UPR activates pathways leading to apoptotic cell death. Therefore, ER-stress plays a significant role in cancer cell apoptosis signaling pathway and its targeting can be a promising anticancer strategy.

We have shown that AdoMet induced ER-stress, through the increase in the spliced form of XBP1 and CHOP levels, in JHU-SCC-011 and HNO210 cell lines. CHOP is one of the key players in the ER-stress-mediated apoptotic pathway and is implicated in the regulation of processes that lead to cell proliferation, differentiation, expression, and energy metabolism. When the expression of CHOP rises sharply, apoptosis is activated. It was also demonstrated that CHOP regulates pro- and anti-apoptotic proteins, increasing the pro-apoptotic Bax/Bcl-2 ratio [40]. Another important target downstream of the ER-stress pathway includes several members of MAPKs family. The MAPK signaling network is known to regulate cell cycle progression and cell survival or death responses, following a variety of stresses [27]. The sustained activation of JNK, ERK and p38 can contribute to both necrotic and apoptotic cell death by regulating the expression of cytotoxic ligands and synthesis of ROS [41]. In addition, stress-activated MAPKs can regulate the intrinsic apoptosis pathway, mediated by mitochondria, by regulating members of the Bcl-2 family.

According to these, we have demonstrated that the pro-apoptotic mechanism of AdoMet on LSCC cell lines is due to its ability to induce ER-stress and to activate UPR by increasing CHOP levels, phosphorylation, and the activation of JNK, ERK and p38.

Moreover, there is a mutual relationship between ER-stress and ROS accumulation. ER-stress increases oxidative load and can cause oxidative stress, depending on the severity of ER-stress [40]. On the other hand, the excessive accumulation of ROS in different cell compartments, especially mitochondria, can induce UPR [42]. AdoMet treatment is able to increase ROS production in LSCCs, but the data have shown that the antagonization of ROS by NAC does not protect the LSCC by the cytotoxic effects of AdoMet, confirming that ROS production is downstream of the ER-stress response.

To better understand if AdoMet anti-proliferative activity could be mediated by its regulation on miRNAs, analysis of their expression profile in JHU-SCC-011 cell line has been carried out through miRNA Array CARD, after 72 h of treatment with 300 μM AdoMet. The results showed the presence of different deregulated miRNAs in cells treated with the sulfonium compound, compared to the control; most of them have targets involved in cancer-related processes, such as proliferation, migration and death pathways. Among these miRNAs regulated by AdoMet, miR-888-5p was validated through qRT-PCR analysis in both LSCC lines. MiR-888-5p has been recently identified as a potent oncomiR in biological processes of prostate, breast, hepatocarcinoma, colorectal and lung cancer [32,43,44,45].

It has been shown that the overexpression of miR-888-5p significantly increases the proliferation and migration of prostate cancer cells [43] and hepatocellular carcinoma, where it promoted cancer growth by decreasing p53 level and induced migration and invasion by targeting SMAD4, leading to the modulation of the main EMT markers [46].

EMT is one of the key molecular steps in the process of tumor progression, enabling cells to lose epithelial characteristics and acquire motile mesenchymal traits. Several pieces of evidence have highlighted the involvement of miR-888-5p in regulating both EMT and cancer cell migration by inhibiting the adherens junction pathway and by targeting E-cadherin expression [43]. Finally, the overexpression of miR-888-5p promoted the invasion and migration of lung adenocarcinoma A549 cells by targeting E-cadherin and tissue inhibitor of metalloproteinase 2 [47].

Nevertheless, no data exist about the roles of miR-888-5p in the clinical pathological correlations and biological functions in laryngeal tumorigenesis.

In the present study, we demonstrated that AdoMet strongly reduced the level of the oncogenic miR-888-5p in JHU-SCC-011 and HNO210 cells. AdoMet and the miR-888-5p inhibitor combination potentiated the pro-apoptotic effects and increased the capability of AdoMet to reduce LSCC migration, suggesting that AdoMet action can be mediated by its regulation on miRNA-888-5p level.

MiRNA recognizes their regulatory targets through base pairing, and computational methods have been invaluable for identifying these targets. Through the use of the bioinformatic software, we identified potential miRNA–mRNA–protein interactions. The most relevant and interesting for our studies were the following: (i) FAS-Receptor, a death receptor on the surface of cells that leads to programmed cell death; (ii) SLC25C, a mitochondrial transporters associated by the input of AdoMet in mitochondrial compartment; (iii) TIMP-2 and TIMP-4, natural inhibitors of the matrix metalloproteinases; (iv) MYCBP, an activator of c-Myc and CDH1; (v) E-cadherin, a regulator of cell adhesion. Interestingly, among them, only two genes were affected by the AdoMet and miR-888-5p inhibitor combined treatment: MYCBP and CDH1.

CDH1 is a gene located on chromosome 16q22.1, encoding E-cadherin. This protein belongs to the cadherin family, which is involved in cell differentiation and the maintenance of normal architecture of epithelial. The loss of epithelial shape is associated with a down-regulation or complete absence of E-cadherin, which has been found in many types of human cancer and this is linked with the impairment of cell adhesion and increased invasiveness through EMT and is correlated with poor prognosis of tumor [32,45].

Specifically, in cancer cells, the low expression of E-cadherin causes abnormal morphogenesis and architecture of epithelial tissues and loss of contact inhibition leading to uncontrolled growth and invasion of adjacent tissues. The normal phenotype is restored when malignant epithelial tumor cells are transfected with wild-type CDH1 [48].

The obtained results showed a downregulation of CDH1 in LSCC by AdoMet and miR-888-5p inhibitor treatment with a consequent decrease in cell migration.

The protein encoded by MYCBP gene binds to the N-terminus of the oncogenic protein c-Myc, enhancing the ability of c-Myc to activate E box-dependent transcription. Although the oncogenic function of Myc is well known, its role as an apoptotic driver, which appears to be intrinsic, is still controversial and not completely studied. Thereby, it has been established that the activation of c-Myc strongly enhances cancer cell sensitivity to apoptosis and represses the expression of several genes involved in the regulation of cell adhesion, motility and invasiveness [49,50].

Several cytotoxic compounds, currently used in cancer treatment, impair tumor cell viability and trigger apoptosis in a c-Myc-dependent manner on a wide variety of cell lines. Cerquetti and colleagues have highlighted that the good response to paclitaxel chemotherapy is correlated to c-Myc overexpression in adrenocortical cancer cell lines, resulting in a strong perturbation of cell cycle followed by apoptotic cell death [50]. Moreover, several manuscripts reported the interaction between c-Myc and UPR signaling, highlighting the direct and indirect regulation operated by c-Myc on this process. Babcock et al. have demonstrated that the c-Myc-dependent induction of UPR enhances bortezomib capability to induce CHOP expression, as well as apoptosis, in tuberous sclerosis complex-null cells, and reversed the ability of rapamycin to prevent the activation of the death pathway [51]. Elucidating the mechanisms underlying c-Myc pro-apoptotic role may represent an effort to optimize the treatment of tumors with Myc deregulation.

Our data strongly support this new role of c-Myc. In fact, the pro-apoptotic effect of AdoMet could be mediated by its indirect action on c-Myc expression, as confirmed by the increase in the apoptotic percentage after the combined treatment with the sulfonium compound and miR-888-5p inhibitor. In addition, the link between c-Myc and UPR shows that AdoMet is able to induce apoptosis in LSCC by enhancing ER stress, and that this mechanism may be mediated by its capability to downregulate miR-888-5p.

## 4. Materials and Methods

### 4.1. Materials

Propidium iodide (PI), 3-(4,5-dimethylthiazol-2-yl)-2,5-diphenyltetrazolium bromide (MTT), radioimmunoprecipitation assay buffer (RIPA buffer), 2′,7′-Dichlorofluorescein diacetate (DCF-DA), *N*-acetyl-l-cysteine (NAC) and CLC were purchased from Sigma-Aldrich (St. Louis, MO, USA). Bovine serum albumin (BSA), fetal bovine serum (FBS), Dulbecco’s modified Eagle’s medium (DMEM), Roswell Park Memorial Institute (RPMI), phosphate-buffered saline (PBS), and trypsin-EDTA were obtained from Gibco (Grand Island, NY, USA). Tissue culture dishes were purchased from Corning (Corning, NY, USA). AdoMet was provided from New England Biolabs, prepared in a solution of 5 mM H_2_SO_4_ and 10% ethanol, filtered, and stored at 4 °C until use. Lipofectamine 2000, mirVANA PARIS Kit, TaqMan-MiRNA Reverse Transcription Kit, Megaplex RT Primers, TaqManPreAmp Master Mix, MegaplexPreAmp Primers, TaqMan Universal PCR Master Mix, 384-well TaqMan MiRNA Array CARD, Opti-minimal essential medium (Opti-MEM) LysoTracker Red DND-99 (LTR), ER Tracker Blue DPX, SYBR™ Green PCR Master Mix and Custom DNA Oligos: CCAAT-enhancer-binding protein homologous protein (CHOP), spliced form of X-box binding protein 1 (sXBP1), unspliced form of X-box binding protein 1 (uXBP1), c-Myc-Binding Protein (MYCBP), cadherin-1 (CDH1)and glyceraldehyde 3-phosphate dehydrogenase (GAPDH) were obtained from Thermo Fisher Scientific (Waltham, Massachusetts, USA). RNasy Mini Kit was obtained from Qiagen (Hilden, Germany). DNase was purchased from Promega (Madison, Wisconsin, USA). VILO Super-Script was obtained from Invitrogen (Carlsbad, California, USA). miRNA-888-5pinhibitor, miRNA-888-5pmimicand scramble negative control (*mir*Vana™ miRNA Inhibitor Negative Control #1) were obtained from Life Technologies (Waltham, MA, USA). An Annexin V-fluorescein isothiocyanate (V-FITC) Apoptosis Detection kit was purchased from eBioscience (San Diego, CA, USA). Monoclonal antibodies (mAb) to caspase 9, poly(ADP ribose)polymerase (PARP), c-Myc, vimentin, matrix metalloproteinase (MMP)-9, β-actin, α-tubulin and polyclonal antibodies (polyAb) to caspase 6, caspase 7, CHOP, extracellular signal-regulated kinase 1/2, (ERK1/2), phospho-Erk 1/2,microtubule-associated protein light chain 3B, (LC3B), mitogen-activated protein kinases 11 (p38), phospho-p38, MMP2were purchased from Cell Signaling Technology (Danvers, MA, USA). Monoclonal antibodies (mAb) to dual specificity phosphatase-1 (Dusp-1), C-Jun N-terminal kinase, (JNK1) and phospho-JNK were purchased from Santa Cruz Biotechnology (Dallas, TX, USA). The monoclonal antibody to E-cadherin was acquired from BD Biosciences (San Josè, CA, USA). Horseradish peroxidase (HRP)-conjugated goat anti-mouse (GxMu-003-DHRPX) and HRP-conjugated goat anti-rabbit (GtxRb-003-DHRPX) secondary antibodies were obtained from ImmunoReagents. Inc. (Raleigh, NC, USA). All buffers and solutions were prepared with ultra-high-quality water. All reagents were of the purest commercial grade.

### 4.2. Cell Cultures and Treatments

The LSCC cell lines, JHU-SCC-011 and HNO210, were provided from the American Type Culture Collection; (ATCC Manassas, VA, USA). JHU-SCC-011 and HNO210 cells were cultured at 37 °C in a 5% CO_2_ humidified atmosphere and grown, respectively, in RPMI or DMEM supplemented with 10% heat-inactivated FBS, 100 U/mL penicillin, 100 μg/mL streptomycin, and 1% L-glutamine. Typically, subconfluent cells were seeded at 1.8 × 10^5^/10 cm and 5.5 × 10^5^/10 cm culture dishes for JHU-SCC-011 and HNO210 cells, respectively. After 24 h, the LSCC cells were treated with 10% FBS fresh medium containing different concentrations of AdoMet for 24, 48, and 72 h. Subsequently, fluctuating cells were recovered from culture medium by centrifugation, whereas adherent cells were collected by trypsinization.

### 4.3. LysoTracker-Red Staining

The LSCC, JHU-SCC-011 and HNO210 cells, were seeded in 6-well plates at the density of 50 × 10^3^ cells/well and 75 × 10^3^ cells/well, respectively, and after 24 h of incubation, the cells were treated with 200 and 300 μM AdoMet for 24 and 48 h. After treatment, LTR was added to each well for 20 min at 37 °C at a final concentration of 0.1 μM in medium. The cells were then washed with PBS and observed by fluorescence microscopy. The fluorescence intensity of the cells was then analyzed by flow cytometry. Then, the cells were detached by incubation with EDTA-trypsin, washed twice with PBS, and collected by centrifugation. The fluorescent emissions were collected as FL1 (linear scale) using BD Accuri™ C6 [52]. At least 20,000 events were recorded in log mode. For the quantitative evaluation of LTR, FlowJo software was used to calculate median fluorescence intensities (MFI) by the formula (MFI-treated/MFI-control), where MFI-treated is the fluorescence intensity of cells treated with the various compounds and MFI-control is the fluorescence intensity of untreated cells. For each sample, 20,000 events were acquired. Analysis was carried out by triplicate determination in at least three separate experiments.

### 4.4. Determination of ROS by DCF-DA Assay

The LSCC, JHU-SCC-011 and HNO210 cells, were seeded in 6-well plates at the density of 50 × 10^3^ cells/well and 75 × 10^3^ cells/well, respectively, and after 24 h of incubation, the cells were treated with AdoMet 200 µM for 72 h, at 37 °C with or without pretreatment with 5 mM NAC. After treatment, cells were stained with 10 μM DCF-DA for 30 min at 37 °C in the dark. Following incubation, cells were washed twice with PBS, trypsinized, resuspended in PBS, and immediately analyzed with a BD Accuri™ C6 flow cytometer (Becton Dickinson, San Jose, CA, USA). For each sample, 20,000 events were recorded. Analysis was carried out by triplicate determination in at least three separate experiments.

### 4.5. ER Tracker Blue-White DPX Staining

The LSCC, JHU-SCC-011 and HNO210cells, were seeded in 24-well plates at the density of 6.0 × 10^3^ cells/well and at the density of 15 × 10^3^ cells/well, respectively. After 24 h of incubation, the cells were treated with 200 and 300 μM AdoMet for 48 and 72 h. The endoplasmic reticulum (ER) stress was identified by staining cells with the ER-specific dye ER Tracker™ Blue/White 1 µM in a solution of PBS, for 30 min at 37 °C. Then, the cells were washed in PBS and fixed with 4% paraformaldehyde solution, permeabilized with 0.1% TRITON-X/PBS solution, and finally blocked with a 1% BSA/FBS solution for 1 h at room temperature. For positive control, cells were exposed for 16 h to 5 μg/mL tunicamycin. Images were collected under a fluorescence microscope (EVOS FL Cell Imaging System, Thermo Scientific, Rockford, IL, USA) after MOVIOL counterstaining.

### 4.6. Cell Transfections

The LSCC, JHU-SCC-011 and HNO210cells, were seeded in 6-well plates at the density of 75 × 10^3^ cells/well and 10 × 10^4^ cells/well, respectively, to achieve 80% of confluence. After 24 h, cells were transfected with 50 nM miR-888-5p inhibitor, scramble negative control or 50 nM miR-888-5p mimic, diluted in Opti-MEM free medium supplemented, or not (Control), with 300 μM AdoMet, by using Lipofectamine 2000 according to manufacturer’s protocol. Lipofectamine was also used alone as a negative control. After 72 h from transfection, cells were harvested and then subjected to the extraction of the total RNA, preparation of cells lysates and flow cytometry analysis.

### 4.7. MiRNA Detection and Validation by qRT-PCR

Total RNA was isolated from cultured cells treated, or not, with AdoMet 300 μM, by using the mirVANA PARIS Kit, according to manufacturer’s instructions. Subsequently, using the TaqMan MiRNA Reverse Transcription Kit and the Megaplex RT Primers, single-stranded cDNA was synthesized from total RNA samples. The pre-amplified cDNA targets were amplified by DNA polymerase from the TaqMan Universal PCR Master Mix using sequence-specific primers and probes on the 384-well TaqMan miRNA Array CARD. The array was loaded and run on Applied Biosystems Viia7 instrument (Life Techonologies, Carlsbad, CA, USA) by using the default thermal cycling conditions. To validate the results of the Array CARDs, the expression of miRNAs was independently determined by quantitative real-time PCR (qRT-PCR), using TaqMan miRNA Assays that use looped-primer to accurately detect mature miRNAs. qRT-PCR was performed on a ViiA7™ Real-time PCR system (Applied Biosystems, Foster City, CA, USA). To normalize total RNA samples, the small-nuclear-U6 was selected as an appropriate constitutively expressed endogenous control. The relative expression of the transcripts was measured by using ViiA7™Real-Time PCR software (Applied Biosystems, Foster City, CA, USA).

### 4.8. Flow Cytometry Analysis of Apoptosis

Annexin V-FITC was used in conjunction with a vital dye PI to distinguish apoptotic (Annexin V-FITC-positive, PI positive) from necrotic (Annexin V-FITC negative, PI positive) cells. After 72 h, LSCC cells, transfected and/or treated, were detached by incubation with EDTA-trypsin, washed twice with PBS, and collected by centrifugation. Then, the cells were resuspended in 200 μL of Binding Buffer 1X and incubated with 5 μL Annexin V-FITC and 10 μL PI (20 μg/mL) for 30 min at room temperature, as recommended by the manufacturers. The detection of viable cells, early apoptosis, late apoptosis, and necrotic cells was performed by BD Accuri™ C6 flow cytometer (Becton Dickinson, San Jose, CA, USA). For each sample, 20,000 events were recorded. Analysis was carried out by triplicate determination on at least three separate experiments.

### 4.9. RNA Isolation, Reverse Transcription, and qRT-PCR

Total RNA was collected from LSCC cultured cells, treated or not with 200 and 300 μM AdoMet, and transfected or not with 50 nM miR-888-5p inhibitor, alone or in combination with AdoMet 300 μM, according to the manufacturer’s instructions, by using RNeasy Mini Kit (Qiagen, Germantown, MD, USA). RNA was treated with DNase (Promega, Madison, WI, USA) to exclude DNA contamination, and 1 μg total RNA was reverse-transcribed using VILO Super-Script (Invitrogen, Carlsbad, CA, USA). Gene expression assays were performed on a StepOne Thermocycler (Applied Biosystems, Foster City, CA, USA) and the amplifications carried out at 95 °C for 15 min, followed by 40 cycles of three steps consisting of denaturation at 94 °C for 15 s, primer annealing at 60 °C for 30 s, and primer extension at 72 °C for 30 s using SYBR Green PCR MasterMix (Applied Biosystems, Foster City, CA, USA). A melting curve analysis was performed from 70 °C to 95 °C in 0.3 °C intervals. Each sample was performed in triplicate. GAPDH was used to normalize for the differences in the RNA input. qRT-PCR primer sequences are followed: CHOP (Forward 5′-AGAACCAGGAAACGGAAACAGA-3′ and Reverse 5′-TCTCCTTCATGCGCTGCTTT-3′); uXBP1, (Forward 5′-CAGCACTCAGACTACGTGCA-3′ and Reverse 5′-ATCCATGGGGAGATGTTCTGG-3′); sXBP1, (Forward 5′-CTGAGTCCGCAAGCAGGTGCAG-3′ and Reverse 5′-ATCCATGGGGAGATGTTCTGG-3′); GAPDH, (Forward 5′-GGAGTCAACGGATTTGGTCG-3′ and Reverse 5′-CTTCCCGTTCTCAGCCTTGA-3′), CDH1, (Forward 5′-TCCGAAGCTGCTAGTCTGAG-3′ and Reverse 5′-CTCAAGGGAAGGGAGCTGAA-3′), MYCBP (Forward 5′-TCCTTGGTGCAGTTCAGGAT-3′ and Reverse 5′-TCAAACTGGCACACACACAC-3′).

### 4.10. Migration Process Evaluated by Scratch-Wound Assay

The LSCC, JHU-SCC-011 and HNO210 cell lines, were seeded in 6-well plates in the appropriate number until 80% confluence was reached in 24 h and transfected with 50 nM miR-888-5p inhibitor or with scramble negative control, diluted in Opti-MEM free medium by using Lipofectamine 2000 (Thermo Fisher Scientific, Waltham, MA, USA) according to manufacturer’s protocol. Following transfection, the next day the medium was changed and was integrated, or not (Control), with 300 μM of AdoMet. Then, in a sterile environment, a 200 μL pipette tip was used to manually press against the top of the tissue culture plate and a vertical downwards wound was rapidly created through the confluent cellular monolayer. Carefully, medium and cell debris were aspirated away, replaced with 2 mL of complete medium and initial images of the wounds were captured using a microscope (Leica Microsystems GmbH) corresponding to time zero (T0). Following 24 h (T1) of treatment, snapshot images were captured to examine for wound closure. The wound areas of the control and treated cells were quantified using ImageJ software 1.48v (U.S. National Institutes of Health, Bethesda, MD, USA).

### 4.11. Protein Extraction and Western Blot Analysis

JHU-SCC-011 and HNO210 cells, grown at 37 °C for 24, 48 and 72 h after each treatment, were lysed using 100 μL of RIPA buffer. After incubation on ice for 30 min, the samples were centrifuged at 18,000× *g* in an Eppendorf micro-centrifuge for 30 min a 4 °C, and the supernatant was recovered. The protein concentration was determined and compared with BSA standard curve. Equal amounts of cell proteins were separated by sodium dodecyl sulfate-polyacrylamide gel electrophoresis (SDS-PAGE) and electrotransferred to nitrocellulose membranes by Trans blot turbo (BIO-RAD). The membranes were washed in 10 mM Tris-HCl, pH 8.0, 150 mM NaCl, 0.05% Tween 20 (TBST), and blocked with TBST supplemented with 5% nonfat dry milk. Then, membranes were incubated first with different primary antibodies in TBST and 5% nonfat dry milk, washed, and then incubated with HRP-conjugated secondary antibodies. All primary antibodies were used at a dilution of 1:1000; all secondary antibodies were used at a dilution of 1:5000. Blots were then developed using enhanced chemiluminescence detection reagents ECL (Cyanagen, Bologna, Italy) and exposed to X-ray film. All films were scanned by using Image J software 1.48 (U.S. National Institutes of Health, Bethesda, MD, USA).

### 4.12. TCGAbiolinks Package

Clinical data and expression levels of miR-888-5p and MYCBP in HNSC-TCGA cohort, were retrieved and analyzed using the TCGAbiolinks R package [53]. Expression correlation analysis was conducted using the R statistical environment on a subset of patients for whom RNA-seq and miRNA-seq data were both available (*n* = 495).

### 4.13. Statistical Analysis

Experiments were performed at least three times with replicate samples, except where otherwise indicated. Data are expressed as mean ± standard deviation (SD). The means were compared using analysis of variance (ANOVA) plus Bonferroni’s *t*-test. A *p*-value of < 0.05 was considered to indicate a statistically significant result.

## 5. Conclusions

Overall, these data suggest that the sulfonium compound may provide a new approach to ameliorate conventional chemotherapy treatment of laryngeal cancer through the regulation of miR-888-5p expression in LSCC. Altogether, the obtained results provide a new insight into the action mechanism by which AdoMet exerts its bioactivity and highlight the potential of AdoMet in miRNA-based approaches for cancer treatment and open the way for future medicinal chemistry studies for this class of compounds.

## Figures and Tables

**Table 1:** Comparison of fold changes detected in microarrays and by qRT-PCR.

miRNAs	Fold-Changes (Log2)			
	JHU-SCC-011		HNO210	
	Microarray	qRT-PCR	Microarray	qRT-PCR
hsa-miR-888-5p	−2.65	−7.63	Not performed	−1.98
hsa-miR-187	3.80	0.08	Not performed	0.01
hsa-miR-491-3p	5.62	0.06	Not performed	−0.12
hsa-miR-618	−4.8	−0.67	Not performed	0.23

## Supplementary Materials

The following are available online at https://www.mdpi.com/2072-6694/12/12/3665/s1, Figure S1: Effects of AdoMet on autophagic process in LSCC, Figure S2: Effect of AdoMet on ER stress and related UPR in LSCC, Figure S3: Evaluation of miR-888-5p inhibitor impact, Figure S4: Effect of AdoMet and miR-888-5p inhibitor combination on apoptotic process in LSCC, Figure S5: Evaluation of miR-888-5p inhibitor impact on cell migration in LSCC, Figure S6: Effect of AdoMet and miR-888-5p inhibitor combination on cell migration in LSCC, Figure S7: Effect of AdoMet and miR-888-5p mimic on *MYCBP* and *CDH1* expression, Figure S8: Effect of AdoMet and miR-888-5p inhibitor on MYC and CDH1 expression.
