# Supplementary material for: Mi-RNA-888-5p Is Involved in S-Adenosylmethionine Antitumor Effects in Laryngeal Squamous Cancer Cells
Source: Cancers (Basel). 2020 Dec 7;12(12):3665. doi: 10.3390/cancers12123665 (PMC7762311; doi:10.3390/cancers12123665)
Supplement: Supplementary file 1 [file cancers-12-03665-s001.pdf]

Supplementary Metarials: Mi-RNA-888-5p Is Involved in S-Adenosylmethionine Antitumor Effects in Laryngeal Squamous Cancer Cells

Martina Pagano, Laura Mosca, Francesca Vitiello, Concetta Paola Ilisso, Alessandra Coppola, Luigi Borzacchiello, Luigi Mele, Francesca Pia Caruso, Michele Ceccarelli, Michele Caraglia, Giovanna Cacciapuoti and Marina Porcelli

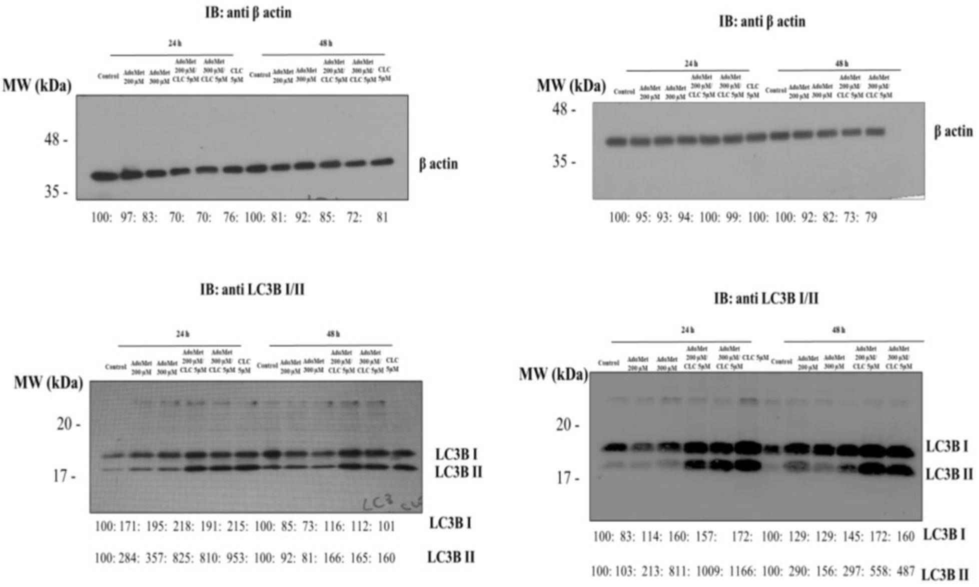

Figure S1. Effects of AdoMet on autophagic process in LSCC**.** The cropped blots are used in the main figure (Figure 1 E and F).

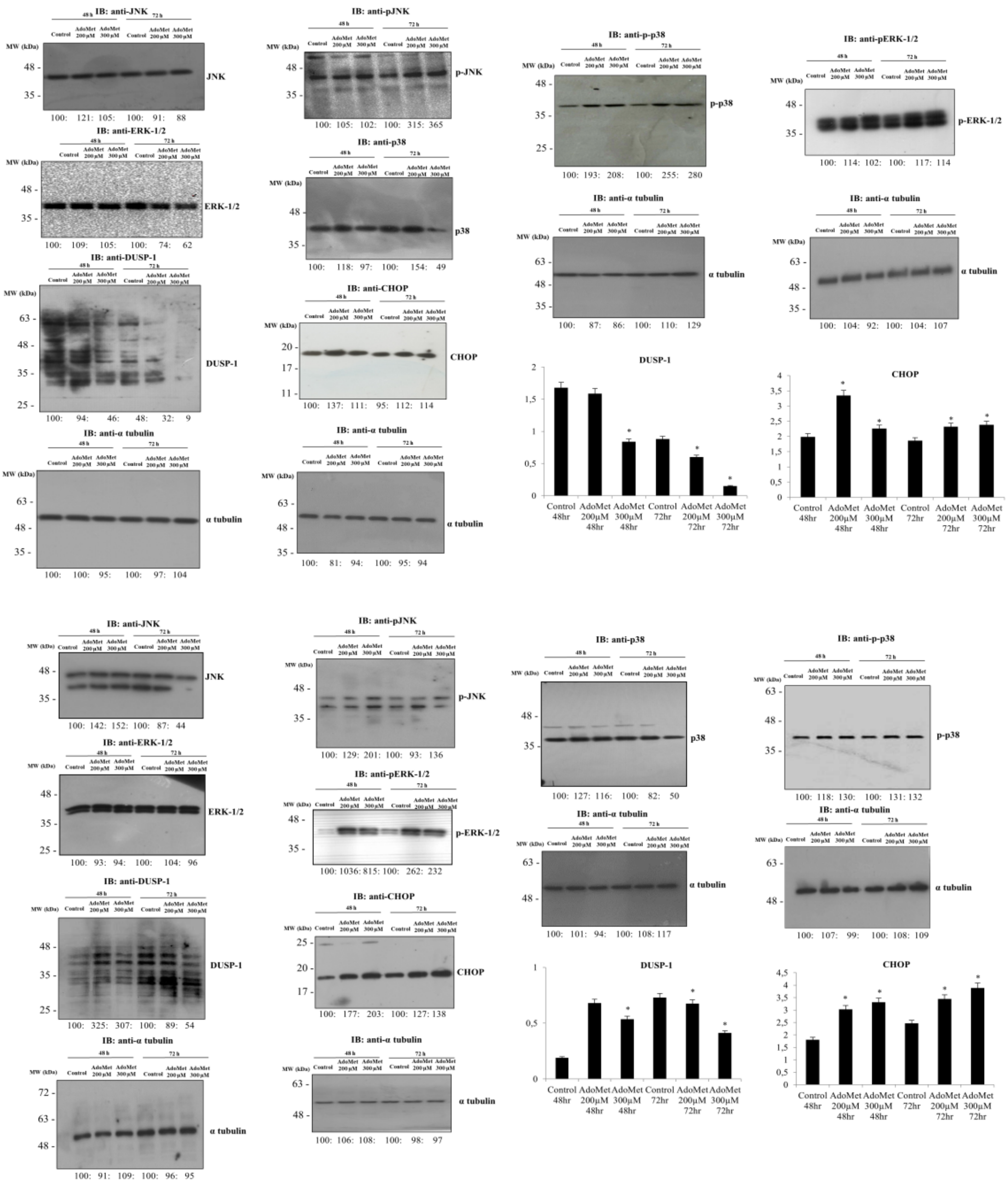

Figure S2. Effect of AdoMet on ER stress and related UPR in LSCC. The cropped blots are used in the main figure (Figure 2 E and F). The graphs shown the densitometric analysis of DUSP-1 and CHOP vs endogenous control. In Figure 2 are reported the densitometric analysis of pERK 1/2/ERK 1/2, pJNK/JNK, p-p38/p38 ratio. The means and SD are shown. *$p < 0.05$ versus control.

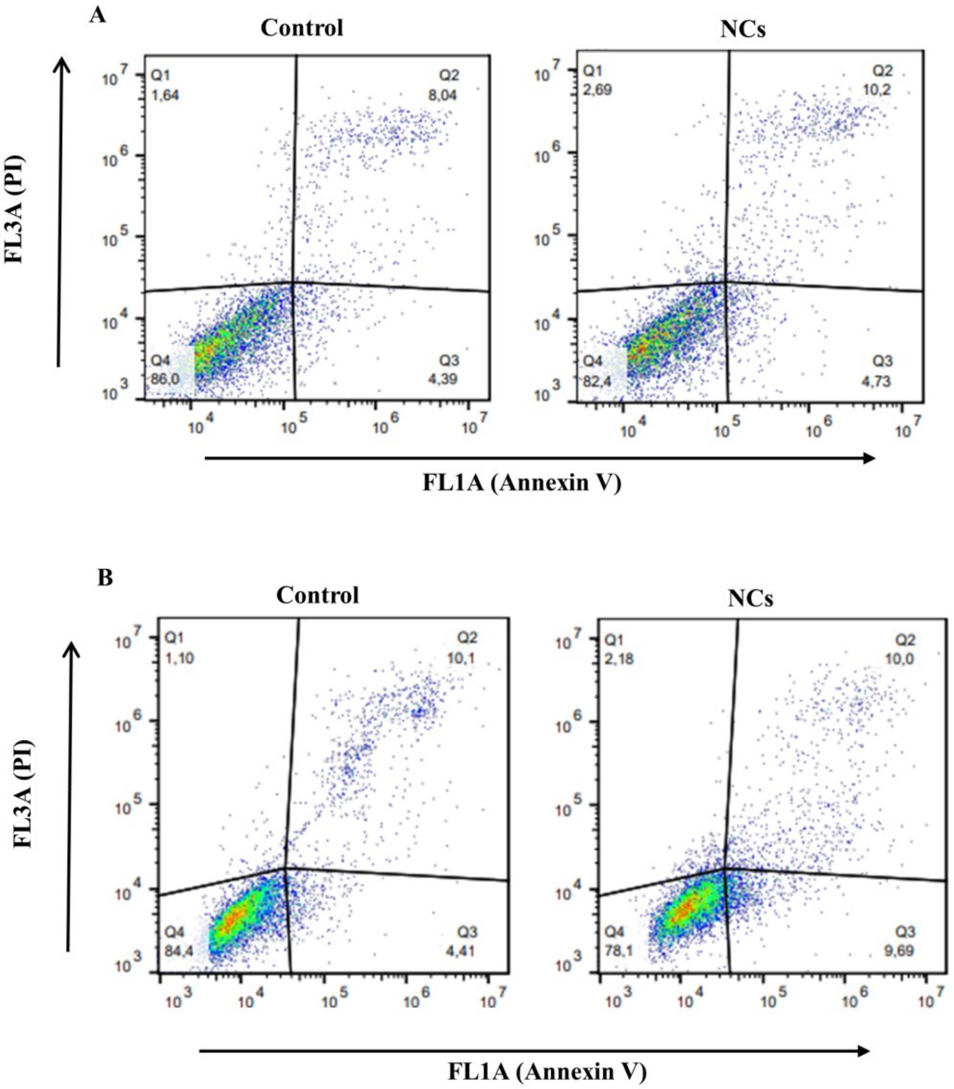

Figure S3. Evaluation of miR-888-5p inhibitor impact. JHU-SCC-011 (**A**) and HNO210 (**B**) cells were transfected with miRNA scramble negative control (NCs) for 72 h, and the apoptosis occurrence was evaluated by FACS analysis.

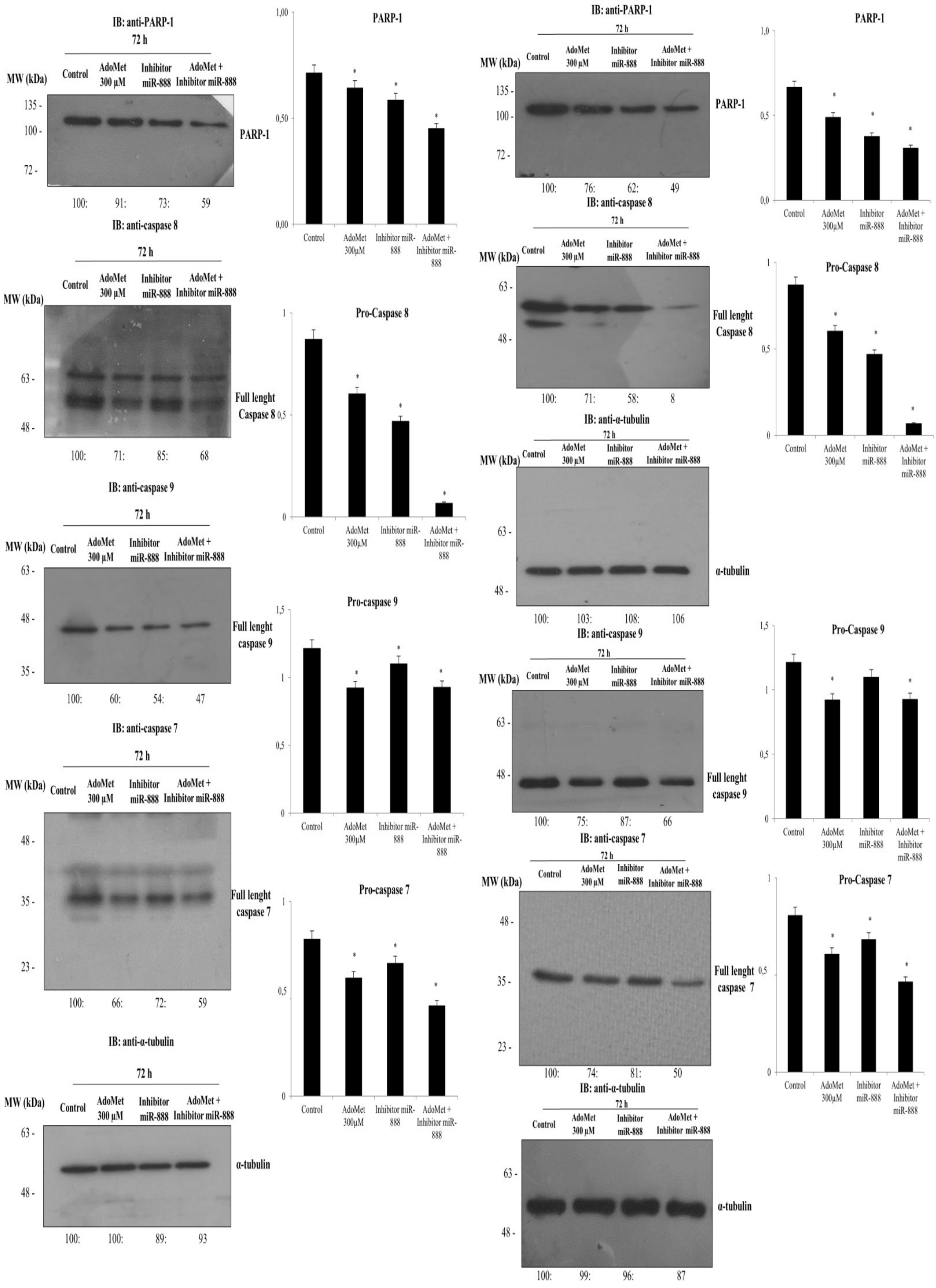

Figure S4. Effect of AdoMet and miR-888-5p inhibitor combination on apoptotic process in LSCC. The cropped blots are used in the main figure (Figure 4 **B** and **C**). The graphs shown the densitometric analysis of each proteins vs endogenous control. The means and SD are shown. *$p < 0.05$ versus control.

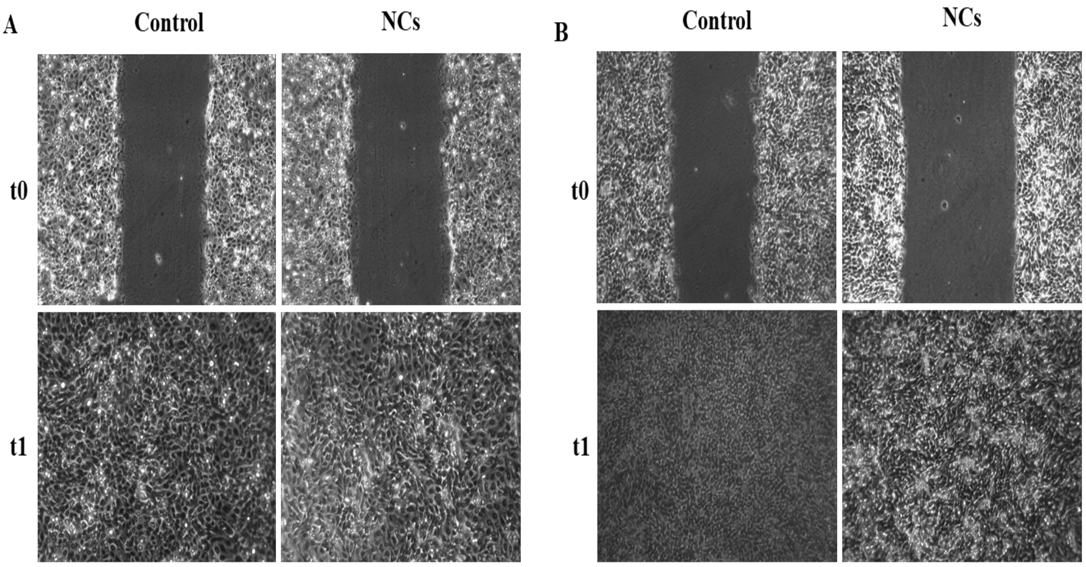

Figure S5. Evaluation of miR-888-5p inhibitor impact on cell migration in LSCC. Confluent monolayers of (**A**) JHU-SCC-011 and (**B**) HNO210 cells treated or not (Control) with scramble negative control (NCs) for 48 hr, were scratched with micropipette tip and snapshot pictures were captured by microscope to check for wound clousure. Pictures of the wounds corresponding to time zero (to) and 24 hr (t1) from the scrape in both cell lines are reported.

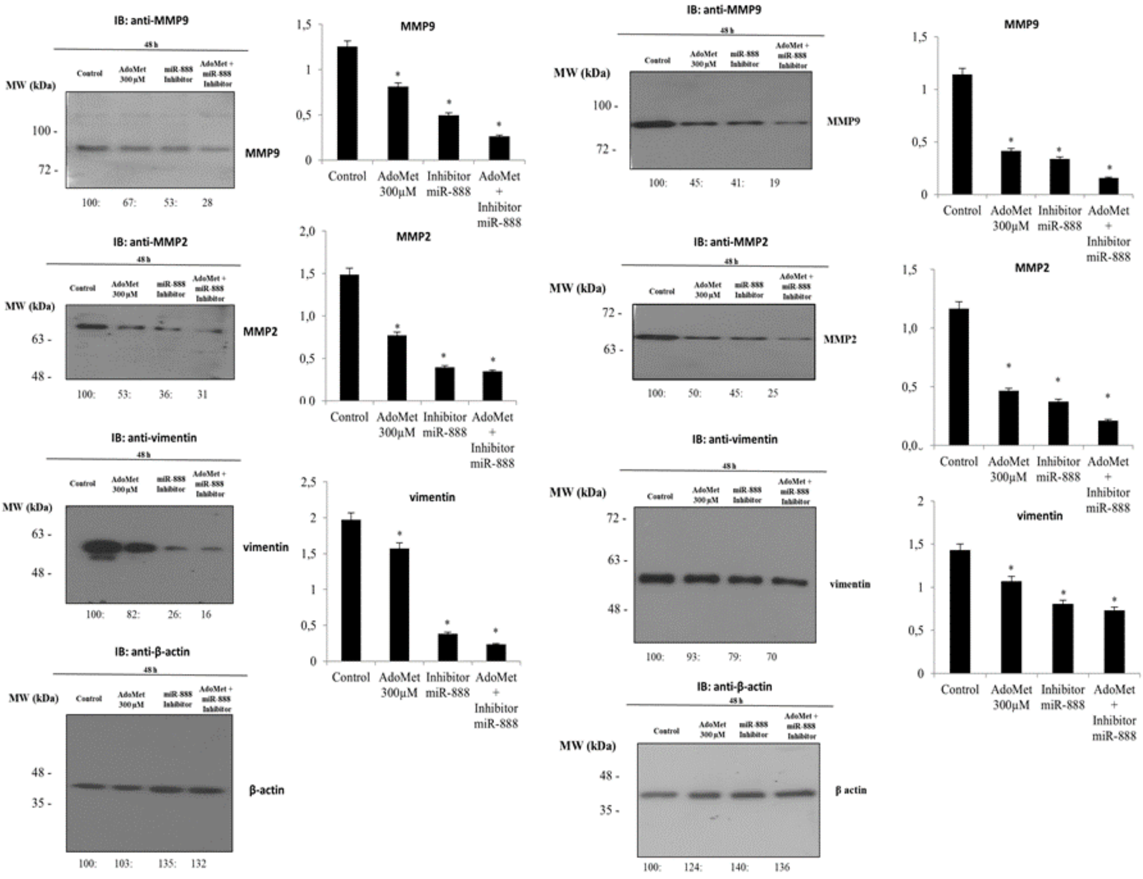

Figure S6. Effect of AdoMet and miR-888-5p inhibitor combination on cell migration in LSCC. The cropped blots are used in the main figure (Figure 5 C and D). The graphs shown the densitometric analysis of each proteins vs endogenous control. The means and SD are shown. *p< 0.05 versus control.

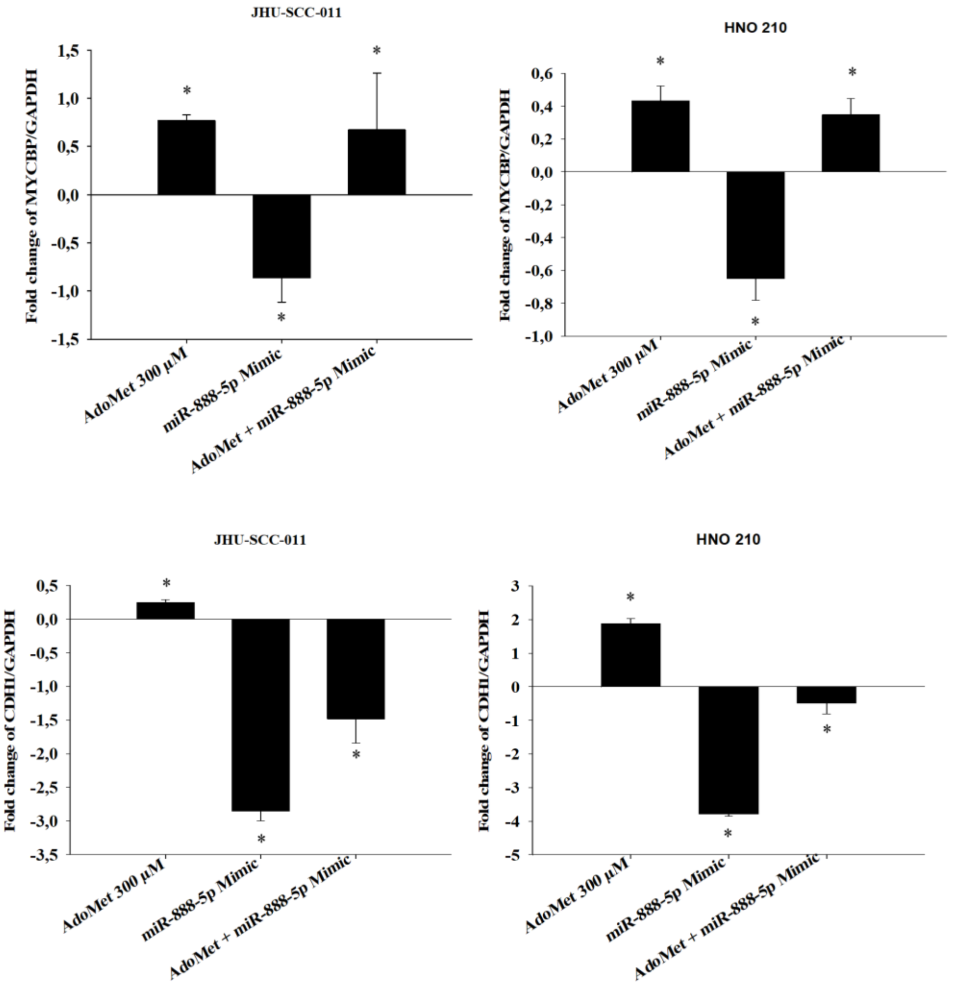

Figure S7. Effect of AdoMet and miR-888-5p mimic on MYCBP and CDH1 expression. (**A–B**) Cells were transfected with miR-888-5p mimic in the presence or not (Control) of 300 µM AdoMet for 48 and 72 hr. Total-RNA of JHU-SCC-011 and HNO210 cells was extracted and cDNA was synthesized by qRT-PCR, to analyze the transcriptional level of predicted target. The graphs show the fold change (**A**) of MYCBP or (**B**) CDH1 in the different experimental conditions normalized to GAPDH mRNA and compared to untreated cells. The means and SD are shown. $^*p < 0.05$ versus control.

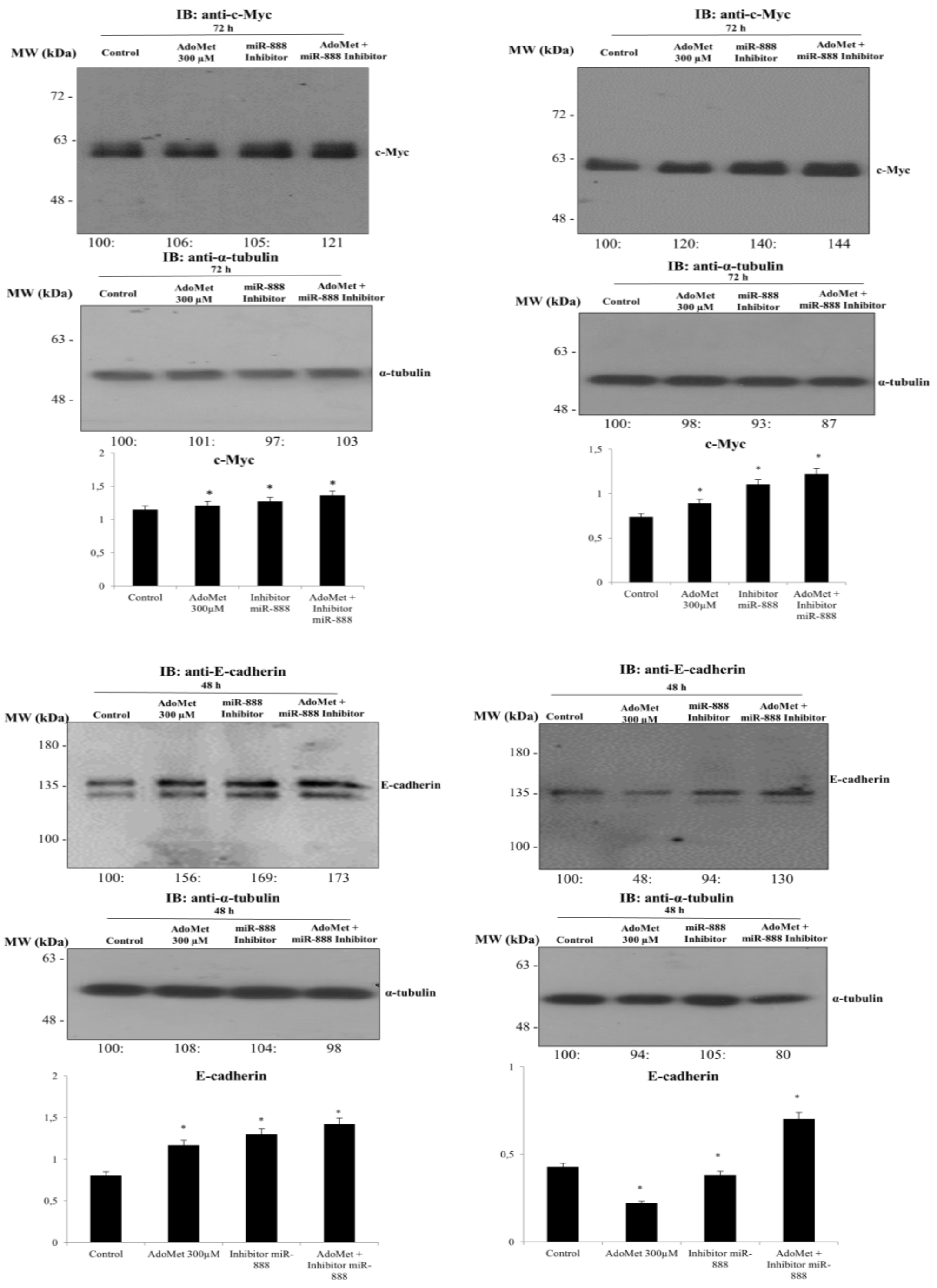

Figure S8. Effect of AdoMet and miR-888-5p inhibitor on MYC and CDH1 expression. The cropped blots are used in the main figure (Figure 6 C). The graphs shown the densitometric analysis of each proteins vs endogenous control. The means and SD are shown. *$p < 0.05$ versus control.
